# A Pin in the Right Bronchus

**Published:** 1890-04

**Authors:** 


A PIN IN THE RIGHT BRONCHUS.—At the meeting of the Surgical Section of the New York Academy of Medicine, January 13th, 1890, Dr. W. T. Bull showed a pin, two inches and three quarters in length, which he had removed from the right bronchus of a child. At the time the pin was swallowed the child had got black in the face, but the spasm had passed off. Pain was complained of for several days, but this was only experienced when the child was raised from a recumbent position. Purulent expectoration was noticed, and the temperature had risen to 102 degrees, with loss of appetite and some vomiting. The speaker had performed tracheotomy, and had luckily found the point of the pin resting at the angle of the incision, while the head of it lay in the right bronchus. The pin had been in the trachea five days.—*New York Medical Journal*, February 8, 1890.

---

A FOOT REFLEX.—Two cases are reported by Dr. Robert Abbe, and one by Dr. V. P. Gilney (*Annals of Surgery*), showing that sometimes a painful flat foot may be entirely relieved by treating and removing an ingrowing toe-nail.—*Medical Record*.

---

LABELS FOR POISONS.—A bill is about to be introduced in the Legislature by Assemblyman Usher, compelling druggists to have printed on the labels of bottles containing poison, the proper antidote.—*Medical Record*.

---

A medical college for colored students has recently been organized at New Orleans, in connection with the New Orleans University.—*Medical Record*.

